# Sex Differences in the Initiation and Progression of Necroptosis Following Kidney Ischemia–Reperfusion Injury

**DOI:** 10.3390/biomedicines13092085

**Published:** 2025-08-27

**Authors:** Minh H. Tran, Colby L. Parris, Catherin Liu, Andrea Oropeza, Carlos Esquivel, Alka Rani, Yingxiang Fan, Liying Fu, Jacentha Buggs, Lei Wang

**Affiliations:** 1 Department of Molecular Pharmacology and Physiology, University of South Florida College of Medicine, Tampa, FL 33612, USA; thm@usf.edu (M.H.T.); colbyparris@usf.edu (C.L.P.); cyl@usf.edu (C.L.); oropezaa@usf.edu (A.O.); carlose1@usf.edu (C.E.); alkarani@usf.edu (A.R.); fying@seas.upenn.edu (Y.F.); 2 Department of Laboratory Medicine and Pathology, Mayo Clinic Arizona, Scottsdale, AZ 85259, USA; fu.liying@mayo.edu; 3 LifeLink of Florida, Tampa, FL 33619, USA; jacentha.buggs@lifelinkfound.org

**Keywords:** sex differences, necroptosis, ischemia, acute kidney injury

## Abstract

**Background:** Ischemia–reperfusion injury (IRI) is a major contributor to acute kidney injury (AKI). While the precise mechanisms of AKI are still incompletely defined, extensive evidence highlights tubular cell injury and death as key factors in its development. Necroptosis has recently emerged as a critical pathway in the pathogenesis of ischemia–reperfusion-induced AKI (IR-AKI). Although sex differences in susceptibility to IR-AKI have been reported, it remains unclear whether there are sex differences in necroptosis dynamics and whether these differences underlie the observed sexual dimorphism in kidney IRI. This study aimed to address these questions. **Methods:** male and female C57BL/6 J mice were subjected to AKI via ischemia induced by bilateral renal pedicle clamping for 18 min at 37 °C. Plasma, urine, and kidney samples were collected at 0 h, 3 h, 6 h, 12 h, 24 h, 48 h, and 72 h post-reperfusion. Kidney injury and function were assessed by measuring plasma creatinine (PCr), blood urea nitrogen (BUN) levels, and histological damage (PAS and cleaved caspase3 staining). Necroptosis activation was assessed by quantifying phosphorylated forms of key markers: p-RIPK1 and p-MLKL. To explore the role of sex hormones in regulating necroptosis dynamics, ovariectomized female mice were subjected to the same IR-AKI protocol, and their kidney injury and functional outcomes were compared with those of intact counterparts. **Results:** The PCr was 0.35 ± 0.04 and 0.32 ± 0.06 mg/dL for males and females, respectively, at 3 h of IR. The levels exponentially increased to 2.05 ± 0.18 at 48 h post-reperfusion in the males but only gradually to 0.94 ± 0.13 mg/dL in females. Necroptosis activation began as early as 3 h post-IR in males but was delayed until ~6 h in females. Males exhibited stronger and more sustained necroptosis activation than females, showing elevated phosphorylation levels of pRIPK1 and pMLKL in Western blot. Female sex hormone deficiency exacerbated the female response to IR-induced injury, which reduced the sex difference in the dynamic of the necroptotic activation and subsequent kidney injury. To our knowledge, this is the first study to characterize sex differences in the initiation and progression of necroptosis and subsequent injury in a mouse model of IR-AKI. **Conclusions:** Our findings reveal distinct temporal patterns of programmed cell death between sexes. Necroptosis-targeted therapies require early intervention in males, which can be delayed in females after IR-AKI, highlighting the need for sex-specific therapeutic windows.

## 1. Introduction

Ischemia reperfusion injury (IRI) is one of the leading causes of acute kidney injury (AKI), occurring when the blood supply to an organ is temporarily obstructed and then restored. IRI occurs during kidney transplantation, severe blood loss, circulatory arrest, or hypotensive shock [1]. Incomplete recovery from IR-AKI can lead to progressive and irreversible damage, ultimately contributing to the development of chronic kidney disease (CKD). Renal IRI causes significant patient morbidity and mortality, contributing to increasing healthcare costs and prolonged medical assistance to the patients [2].

AKI is commonly associated with disruptions in cell homeostasis, resulting from an imbalance between the loss of kidney parenchymal cells and inadequate regeneration or the recruitment of maladaptive cell types. This mismatch between cell death and repair contributes to tubular atrophy and capillary rarefaction [3,4]. These structural changes, in turn, amplify inflammation by facilitating immune cell infiltration and enhancing the release of pro-inflammatory mediators from dying or stressed cells [5]. Therefore, modulating cell death pathways may offer a potential therapeutic strategy for preventing, treating, or enhancing recovery from kidney disease by preserving kidney parenchymal cells and reducing inflammation.

Among several cell death forms, necroptosis, a regulated necrosis format, has recently been identified to play an important role in several models of kidney injury, including IR-AKI. The necroptosis pathway is characterized as a caspase-8–independent nonapoptotic cell death with receptor-interacting protein kinase (RIPK1 and RIPK3) and MLKL (mixed lineage kinase domain-like protein) activation and subsequent formation of necrosome via their RIP homology interaction motifs (RHIMs) [6,7,8,9,10]. Phosphorylated MLKL assembles into pro-necroptotic oligomers, which then translocate to the plasma membrane, compromising the cell’s structural integrity. Unlike RIPK1- and caspase-8-mediated apoptosis, where the cell membrane remains intact, necroptosis culminates in the breakdown of the plasma membrane. This form of cell death is markedly pro-inflammatory because it leads to the liberation of intracellular damage-associated molecular patterns (DAMPs), inflammatory cytokines, and chemokines [11]. Numerous preclinical investigations, employing both genetically modified mice and specific necroptosis inhibitors, highlight the significant role of necroptosis in the development of IR-AKI [12,13,14].

There is evidence of sexual dimorphism in renal injury in both clinical and animal models, with studies suggesting that female sex hormones may have a protective role, while male hormones could have detrimental effects in renal IRI [15,16]. However, not much is known about whether there are any sex differences in the onset or progression of necroptosis and whether these differences contribute to the sexual dimorphism in IR-AKI.

Therefore, this study aims to uncover the potential sex-dependent differences in the dynamics of RIPK1-dependent necroptosis progression over time in IR-AKI by examining the alters of RIPK1, RIPK3, MLKL, phosphorylated RIPK1 (p-RIPK1), phosphorylated MLKL (p-MLKL), and their association with the morphological and functional outcomes. To further understand the influence of ovarian female hormones, i.e., estrogen and progesterone, on this process, females with ovariectomy were induced with IR-AKI, and the dynamics of necroptosis markers were investigated over time. Elucidating these differences may provide valuable insights into the pathogenesis of IR-AKI and its progression in different sexes, potentially informing the development of sex-specific therapeutic strategies to block necroptosis and ameliorate kidney disease.

## 2. Methods

### 2.1. Study Design and Animals

**Figure 1:**
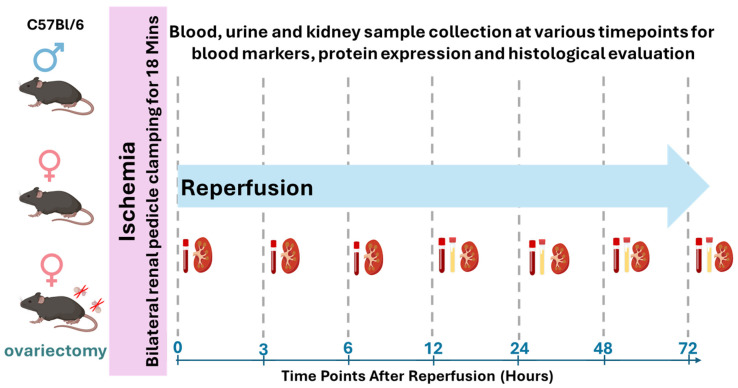
Schematic diagram of the study design. AKI was induced by bilateral renal pedicle clamping for 18 min followed by reperfusion for various time periods. A total of 40 male and 80 female C57 BL/6 J mice were allocated to male sham, male AKI, female sham, female AKI, ovariectomy AKI sham, and ovariectomy AKI groups (*n* = 5/group at each time point).

Male and female C57 BL/6 J mice (12 weeks old, weighing 25–32 g) were obtained from Jackson Laboratory (Bar Harbor, ME, USA). After arrival, mice were housed in a temperature-controlled environment under a 12:12 h light–dark cycle with ad libitum access to food and water for 1 week prior to experiments. Both male and female mice were randomly divided into Sham and IR-AKI groups. AKI was induced by IR with varying reperfusion durations, i.e., 3 h, 6 h, 12 h, 24 h, 48 h, and 72 h for every experiment group, as shown in Figure 1. Sham surgery was performed on respective control groups of mice. A separate group of female mice underwent ovariectomy before AKI induction. Blood, urine, and kidney samples were collected at multiple time points to assess the severity of kidney injury, histopathological changes, and the expression of necroptosis markers.

All animal procedures were approved by the Institutional Animal Care and Use Committee at the University of South Florida and the National Institutes of Health’s Guide for the Care and Use of Laboratory Animals. Animals were euthanized as needed according to the guidelines set forth by the American Veterinary Medical Association (AVMA). All chemicals were purchased from Sigma-Aldrich (St. Louis, MO, USA) unless otherwise indicated.

### 2.2. IR-AKI Induction

IR-AKI was induced in both male and female mice by bilaterally clamping the renal pedicles for 18 min as previously described [17,18]. Briefly, mice were anesthetized with intraperitoneal pentobarbital (50 mg/mL), and body temperature was maintained at 37 °C using a heating pad and lamp. A midline abdominal incision was made to expose the kidneys. Bilateral renal pedicles were carefully dissected and occluded using vascular clips (AESCULAP, Tuttlingen, Germany) for 18 min to induce ischemia. After the ischemic period, the clips were removed to allow reperfusion. The abdominal wall was closed in layers using absorbable Vicryl sutures for the muscle layer and monofilament nylon sutures for the skin. Mice were given 0.4 mL warm saline and monitored on a heating pad until full recovery of consciousness. Sham-operated animals underwent identical surgical procedures and anesthesia duration, except without renal pedicle clamping.

### 2.3. Ovariectomy

Surgical removal of ovaries in randomly selected female mice was performed as previously described [19,20]. Briefly, mice were anesthetized using 2% isoflurane and positioned laterally on the surgical station. A ~1 cm incision was made midway between the hips and ribs. The ovary and uterine horn were identified and gently exteriorized through the incision. The junction between the ovary and uterine horn was located, and the ovary was severed using a cautery tool. The muscle wall and skin were then closed. The same procedure was repeated on the contralateral side to remove the second ovary.

### 2.4. Assessment of Kidney Injury and Function

Plasma creatinine (PCr) and blood urea nitrogen (BUN) levels were measured in the blood samples collected via tail vein puncture using heparin-coated capillaries. Plasma BUN was quantified using the Urea Nitrogen Colorimetric Detection Kit (Invitrogen, Carlsbad, CA, USA) according to the manufacturer’s instructions. PCr levels were measured by High-Performance Liquid Chromatography (HPLC) at the O’Brien Core Center for AKI Research at the University of Alabama.

### 2.5. Immunoblotting

The expression level of necroptotic markers at different time points of reperfusion was evaluated with Western blot as described previously [21,22,23]. Protein was extracted from kidney tissues, separated by SDS-PAGE, and transferred onto membranes. The membranes were then blocked with 5% BSA or non-fat dry milk (NFDM) in TBST, followed by overnight incubation at 4 °C with primary antibodies, including RIPK1 (Cell Signaling, Danvers, MA, USA; 3493, 1:1000), RIPK3 (Cell Signaling, 95702, 1:1000), MLKL (Cell Signaling, 37705, 1:1000) and its phosphorylated forms p-RIPK1 (Cell Signaling, 31122, 1:1000) and p-MLKL (Abcam, Cambridge, UK; 196436, 1:1000). Afterward, the membranes were incubated with secondary antibodies and detected by enhanced chemiluminescence (HRP-conjugated anti-rabbit IgG secondary antibody; ECL, Pierce) or the LI-COR Odyssey^®^ CLx imaging system (LI-COR IRDye^®^ 680 RD or 800 CW goat anti-rabbit; LICOR Biotechnology, Lincoln, NE, USA). Quantification of Western blot images was done by making density plots for each gel in ImageJ (version 1.53) software and normalizing it to the corresponding GAPDH protein expression densities. The protein expression was calculated as the ratio of protein of interest to GAPDH.

### 2.6. Histological Analysis

Kidneys were harvested, fixed in 4% paraformaldehyde, embedded in paraffin, sectioned at 4 µm thickness, and subjected to periodic acid–Schiff (PAS) staining and immunofluorescent staining for cleaved caspase-3 (cCasp3) (Cell Signaling, #9661, 1:400) and p-MLKL (Abcam, ab196436, 1:100) as previously described [21,22,23]. Renal injury was assessed by quantifying the percentage of necrotic tubules showing any signs of necrosis (e.g., loss of brush border, cell blebbing, tubular dilation, sloughing) in PAS-stained sections. The cCasp3 and the p-MLKL index were calculated as the ratio of the positively stained area to the total kidney area. For statistical analysis, at least 10 randomly selected visual fields per specimen were captured at 200× magnification using a Carl Zeiss Axio Observer 7 Apotome System (Oberkochen, Baden-Württemberg, Germany). Quantification of positive staining was performed using Fiji/ImageJ (version 1.53) software.

### 2.7. Real-Time PCR

The inflammation response to IR-AKI was assessed by quantifying the expression of pro-inflammatory cytokines using RT-PCR. Total RNA was extracted from fresh kidney tissue using Trizol reagent (Invitrogen, Carlsbad, CA, USA) and used for real-time PCR analysis as previously described [22,24,25]. The cDNA was synthesized from RNA using the High-Capacity cDNA Reverse Transcription Kit (Applied Biosystems, Foster City, CA, USA) following the manufacturer’s instructions. Quantitative PCR was performed using the CFX96 Real-Time Detection System (Bio-Rad, Hercules, CA, USA) with iQ SYBR Green Supermix (Bio-Rad, Hercules, CA, USA). The primer pairs used for amplification of the interested genes were TNF-α-Fwd: 5′-AGCCCCCAGTCTGTATCCTT-3′, TNF-α-Rev: 5′-CTCCCTTTGCAGAACTCAGG-3′, and IL-1β-Fwd: 5′-GCAACTGTTCCTGAACTCAACT-3′, and IL-1β-Rev: 5′-ATCTTTTGGGGTCCGTCAACT-3′, purchased from Integrated DNA Technologies (IDT, Coralville, IA, USA). The β-actin was used as an internal control. Gene expression levels were calculated using the 2^−ΔΔCt^ method. All reactions were conducted in triplicate, and data were normalized to β-actin expression.

### 2.8. Statistical Analyses

All data are presented as mean ± standard deviation (SD), except where otherwise noted. For renal injury evaluation post-IRI, repeated measures two-way ANOVA was used to examine the effects of time (within-subject factor), sex, and AKI treatment (between-subject factors). Both main effects and interaction effects (e.g., time × sex) were assessed. Tukey’s or Sadak’s multiple comparisons tests were applied to determine statistical significance. The 95% confidence intervals were calculated and reported when appropriate. Necroptosis marker levels post-IRI were compared across groups using one-way ANOVA followed by Tukey’s multiple comparisons tests. Statistical significance was set at *p* < 0.05. All analyses were performed using GraphPad Prism (version 9.0 h).

## 3. Results

### 3.1. Sex Differences in IR-Induced Injury and Morphological Changes

**Figure 2:**
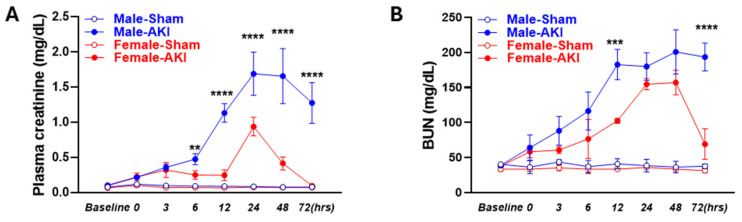
Sex-specific dynamics of IR-induced AKI. (**A**) Plasma creatinine and (**B**) blood urea nitrogen (BUN) levels at various timepoints post-IR in male as compared to female mice. (** *p* < 0.01, *** *p* < 0.001, and **** *p* < 0.0001, Repeated measures two-way ANOVA was used to evaluate the effects of time, sex, and AKI treatment, with time as a within-subject factor and sex and treatment as between-subject factors. Significant main effects and time × sex interactions were observed. Tukey’s multiple comparisons were performed at each time point. The 95% confidence intervals were calculated for males vs. females within each timepoint and are summarized in the Supplemental Table. *n* = 5 mice/group).

As shown in Figure 2A,B, there were no differences in the basal PCr and BUN levels in both male and female mice. Following the induction of IR, both male and female mice exhibited significant increases in plasma creatinine and BUN, with peak concentrations observed between 24 and 48 h post-reperfusion. These circulating markers began to decline after 48 h post-reperfusion, indicating the onset of renal recovery. Notably, male mice displayed substantially higher levels of both plasma creatinine and BUN compared to females beyond 6 h, with male PCr levels 80–450% higher than females at 12–72 h, reflecting greater injury severity. Moreover, peak injury persisted till 48 h in males, whereas females exhibited an earlier trend toward recovery. BUN levels showed a similar temporal and sex-dependent pattern.

**Figure 3:**
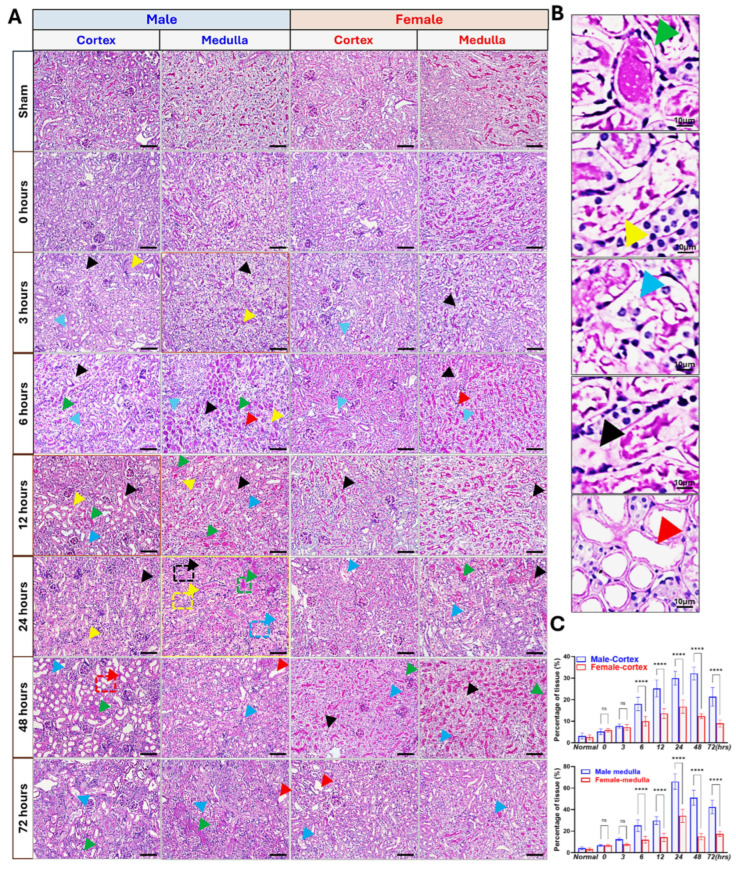
PAS staining reveals dynamics of IR-induced AKI. (**A**). PAS staining (200× magnification, scale bar = 50 μm) showing morphological evidence of sex differences in the IR-AKI. (**B**). Enlarged insets highlight representative injury features from panel A. Black arrows indicate epithelial cell blebbing; yellow arrows, cell sloughing into the lumen; green arrows, casts; blue arrows, brush border disruption; red arrows, empty lumen; (**C**). The percentage represents the estimated area of tubules showing any signs of necrosis (e.g., loss of brush border, cell blebbing, tubular dilation, sloughing). (ns, not significant; **** *p* < 0.0001, Statistical analysis was performed using two-way ANOVA followed by Sadak’s multiple comparisons test. The 95% confidence intervals were calculated within each timepoint and are summarized in the Supplemental Table. *n* = 5/group).

PAS staining revealed acute tubular injury features (epithelial blebbing, nuclear loss, brush border disruption, intratubular cast formation; Figure 3). These abnormalities were more severe in males from 6 h onward, peaking at 24–48 h. The histological findings closely mirrored the biochemical indicators of renal injury.

### 3.2. Sex Differences in IR-Induced RIPK1-Dependent Necroptotic and Apoptotic Markers

**Figure 4:**
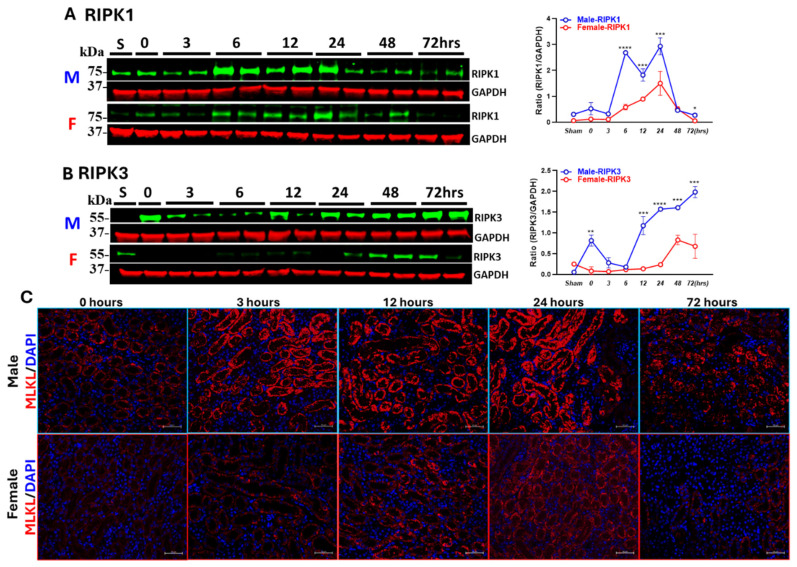
Sex-specific dynamics of necroptotic markers in IR-AKI. Western blot analysis of necroptotic markers in kidney, (**A**) RIPK1 and (**B**) RIPK3 at various reperfusion time points after ischemia. (M-male, F-female) (**C**) Immunofluorescence staining (200× magnification, scale bar = 50 μm) of MLKL in male and female kidney sections. (* *p* < 0.05, ** *p* < 0.01, *** *p* < 0.001, **** *p* < 0.0001; statistical analysis was performed using two-way ANOVA followed by Sadak’s multiple comparisons test; 95% confidence intervals were calculated within each timepoint and are summarized in the Supplemental Table. *n* = 5/group).

Protein was extracted from kidney tissues collected at various reperfusion time points to evaluate changes in necroptotic markers—RIPK1, RIPK3, and MLKL—in both male and female mice. As shown in Figure 4A,B, RIPK1 and RIPK3 proteins were significantly upregulated, which was dynamic across timepoints in both males and females. RIPK1 peaked earlier than RIPK3 in both sexes. However, in male mice, expression of RIPK1 was significantly higher as compared to female mice starting from 3 h post-reperfusion. At peak hours, RIPK1 levels increased ~10-fold in males and ~5-fold in females.

In male mice, RIPK3 expression was upregulated right after ischemia at 0 h, which declined early post-reperfusion, then rebounded after 12 h, peaking at 72 h (~8-fold vs. baseline). In contrast, females exhibited a delayed response, with RIPK3 expression highest at 48 h. The RIPK3 levels were extremely higher in males as compared to females after 12 h post-reperfusion. Similarly, MLKL stain was found to be more prominent in male kidneys as compared to female kidneys at all the time points post-reperfusion (Figure 4C).

### 3.3. Males Exhibit Earlier and More Persistent Necroptosis Activation than Females

**Figure 5:**
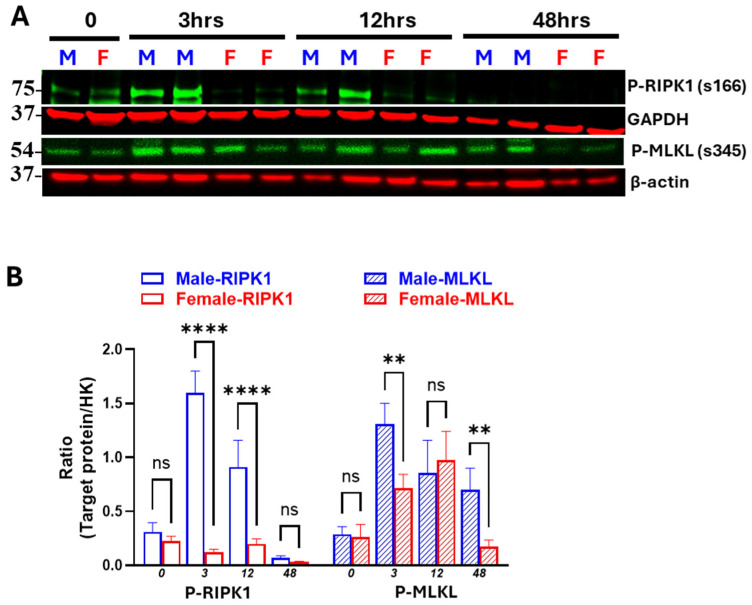
Early and sustained activation of necroptosis in males in IR-AKI. (**A**,**B**) Western blot analysis of the phosphorylated RIPK1 (p-RIPK1) and phosphorylated MLKL (p-MLKL) demonstrating stronger and sustained activation of necroptosis in males than females after IR. (M-male, F-female, ns, not significant, ** *p* < 0.01, **** *p* < 0.0001; statistical analysis was performed using two-way ANOVA followed by Sadak’s multiple comparisons test; 95% confidence intervals were calculated within each timepoint and are summarized in the Supplemental Table, *n* = 5 mice/group).

To assess sex differences in the activation of necroptosis markers following IR, we compared the phosphorylation status of the key initiator p-RIPK1 and the downstream effector p-MLKL between male and female mice using a single Western blot membrane for direct comparison. Representative time points from the early, middle, and late phases post-IR were selected. As shown in Figure 5A,B, both males and females exhibited necroptosis activation in response to IR; however, males showed an earlier onset and a more pronounced activation compared to females. At 3 h post-reperfusion, p-RIPK1 and p-MLKL levels were ~7-fold and ~1.8-fold higher in males than females, respectively. In addition, male mice showed sustained activation, with elevated levels of p-MLKL persisting up to 48 h post-reperfusion. In contrast, female mice exhibited a delayed and attenuated necroptosis response, with markedly lower levels of p-RIPK1 and p-MLKL at most time points as compared to males and near-complete resolution by 48 h.

**Figure 6:**
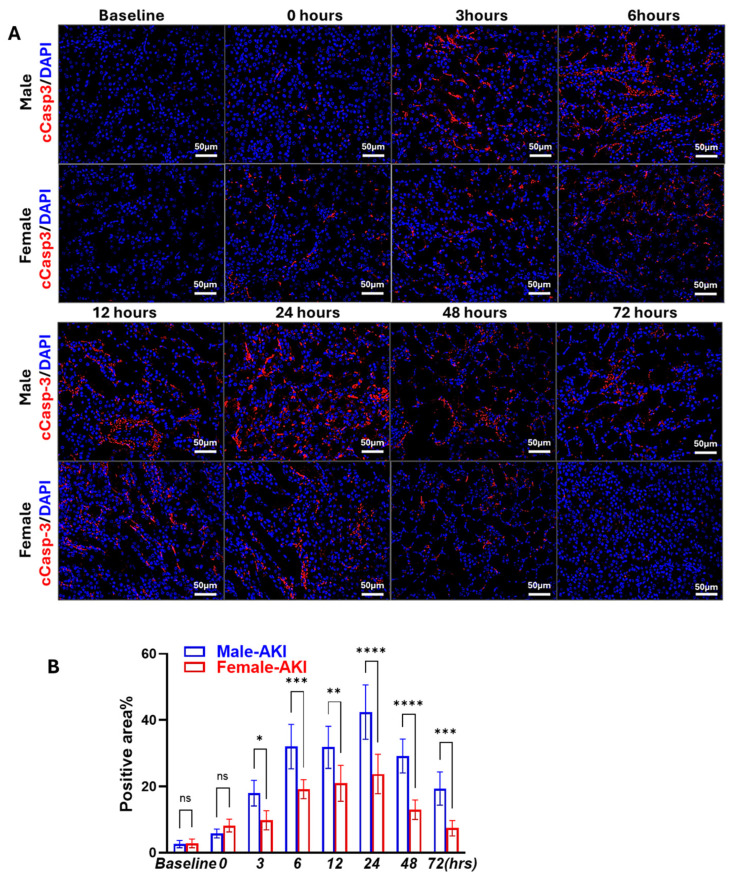
Sex-specific dynamics of apoptosis in IR-AKI. (**A**) Cleaved caspase-3 (cCasp3) staining showing sex differences in the dynamics of apoptosis following IR. (200× magnification, Scale bar = 50 μm, ns, not significant, * *p* < 0.05, ** *p* < 0.01, *** *p* < 0.001, **** *p* < 0.0001; statistical analysis was performed using two-way ANOVA followed by Sadak’s multiple comparisons test; 95% confidence intervals were calculated within each timepoint and are summarized in the Supplemental Table, *n* = 5 mice/group).

To investigate the temporal dynamics of RIPK1-associated apoptosis following IRI, kidney sections collected at various reperfusion timepoints were stained with cCasp3, a key executor of apoptosis. As shown in Figure 6A,B, both male and female mice exhibited time-dependent increases in cCasp3-positive tubular cells, with apoptotic activity becoming more evident after 6 h of reperfusion. Notably, males showed earlier and stronger apoptosis, with cCasp3 significantly elevated by 3 h. In contrast, females showed delayed and attenuated activation of cCasp3, consistent with a sex-dependent modulation of cell death pathways. After 72 h of reperfusion, the expression was negligible in the female kidney sections, showing complete resolution.

### 3.4. Male Mice Showed Earlier and More Persisted Inflammatory Response than Females

**Figure 7:**
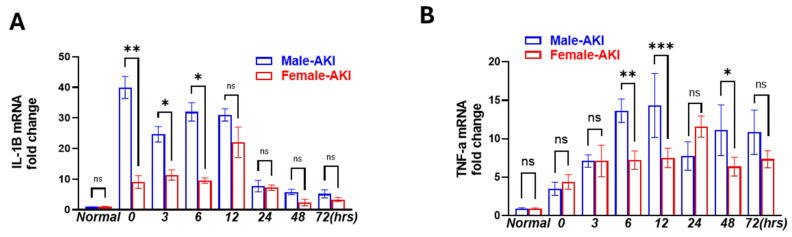
Early and sustained inflammation response in males in IR-AKI. RT-PCR of (**A**) IL-1β and (**B**) TNF-α demonstrating an earlier and more sustained inflammation response in males than females. (ns, not significant, * *p* < 0.05, ** *p* < 0.01, *** *p* < 0.001; statistical analysis was performed using two-way ANOVA followed by Sadak’s multiple comparisons test; 95% confidence intervals were calculated within each timepoint and are summarized in the Supplemental Table, *n* = 5/group).

IR-AKI is characterized by a robust inflammatory response. We observed a significant increase in IL-1β and TNF-α gene expression in both male and female kidneys after IR (Figure 7A,B). Although the levels of both cytokines’ mRNA levels began to decline after 48 h in both sexes, the magnitude of the increase was consistently higher in males. IL-1β mRNA was ~2.5- and 3-fold higher than in females at 3 and 6 h after reperfusion, respectively. TNF-α mRNA was 2- and 1.5-fold higher in males than females at 6, 12, and 48 h. Importantly, the onset of IL-1β and TNF-α markedly elevated immediately after clamp release at 0 h in males. While in females, the peak increase was delayed by 3–6 h. Overall, these findings indicate that males mounted an earlier, stronger, and more sustained inflammatory response than females after IR-AKI.

### 3.5. Removal of Ovarian Hormones Blunts Sex-Specific Differences in Necroptosis Timing and Intensity

**Figure 8:**
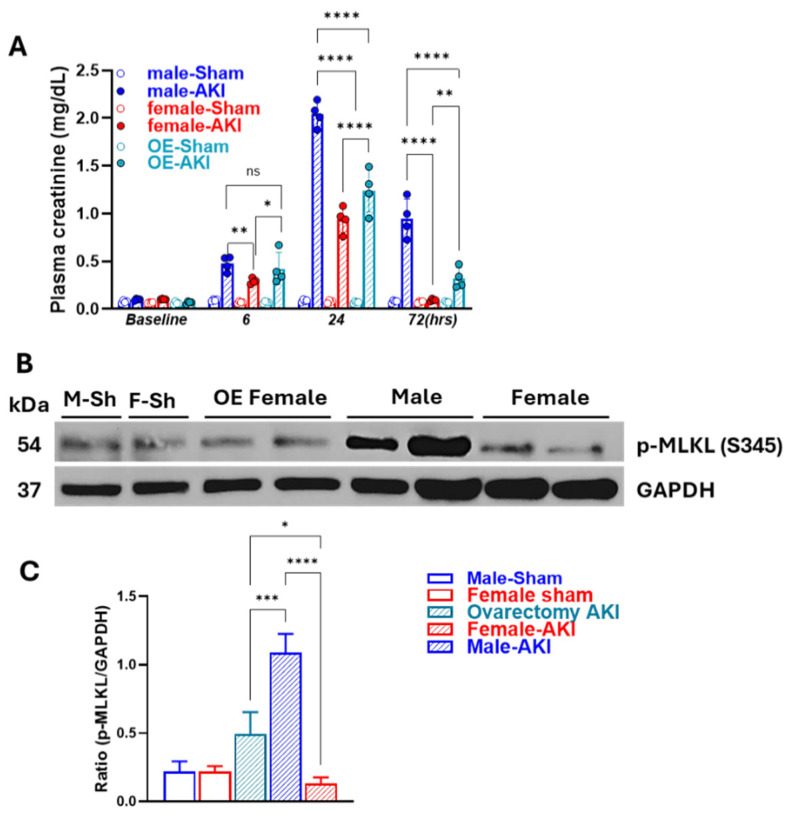
The necroptosis response is exacerbated in ovariectomized females in IR-AKI (**A**) Plasma creatine showing exacerbated kidney injury after IR-AKI in females with ovariectomy compared to females with intact ovaries; Statistical analysis was performed using two-way ANOVA followed by Sadak’s multiple comparisons test; 95% confidence intervals were calculated within each timepoint and are summarized in the Supplemental table. (**B**,**C**) Western blot of phosphorylated MLKL (p-MLKL) demonstrated ovariectomized female mice have higher expression of p-MLKL than intact females at 12 h. (ns, not significant, * *p* < 0.05, ** *p* < 0.01, *** *p* < 0.001,**** *p* < 0.0001; statistical analysis was performed using one-way ANOVA followed by Tukey’s multiple comparisons test; 95% confidence intervals were calculated within each timepoint and are summarized in the Supplemental Table, *n* = 5/group).

To investigate whether female hormones contribute to sex differences in the initiation and progression of necroptosis, we removed the ovaries by ovariectomy. Twenty-one days later, IR-AKI was induced like other groups. As shown in Figure 8A, IR in ovariectomized females exacerbated kidney injury as compared to normal female-AKI mice, as indicated by elevated PCr levels, although these levels did not reach those observed in males. Western blot analysis of p-MLKL at 12 h in ovariectomized females showed higher expression levels as compared to females with intact ovaries, indicating a more aggravated response to IR in the absence of female hormones, albeit to a lesser extent than males (Figure 8B,C).

## 4. Discussion

In this study, we conducted a comprehensive temporal analysis of sex differences in necroptosis activation using a bilateral IRI-AKI mouse model. Our results show that males exhibited earlier onset and sustained activation of necroptotic markers (p-RIPK1 and p-MLKL) compared to females. This heightened necroptotic response in males likely contributes to the more severe renal injury and functional impairment observed. Notably, ovariectomy partially reversed the blunted necroptotic response in females, suggesting that ovarian hormones play a protective role by delaying and attenuating necroptosis activation.

Preclinical and clinical studies consistently show greater male susceptibility to stress-induced kidney injury and functional impairment than females [26,27]. In our moderate-to-severe AKI models, we performed a detailed assessment of injury markers over 0–72 h of reperfusion. Our findings demonstrated that males develop kidney injury earlier and reach higher peak levels of injury markers than females. Furthermore, the recovery phase was significantly delayed in males, indicating prolonged injury resolution compared to their female counterparts.

Necroptosis, a regulated form of necrotic cell death, plays a critical role in the pathogenesis of kidney IRI [28,29,30]. Among the necroptotic pathways, the RIPK1-dependent cascade is the most extensively characterized. It involves sequential activation of RIPK3 and MLKL, culminating in the formation of the necrosome complex [31,32]. While p-MLKL is considered a hallmark of necroptosis, it mostly depends on upstream signaling through RIPK3 and RIPK1. Our analysis revealed early upregulation of RIPK1 and RIPK3 right after ischemia, likely due to ATP depletion and TNF-α signaling. These markers declined transiently upon reperfusion—possibly due to metabolic restoration—before rising again between 12 and 24 h, coinciding with a robust increase in MLKL activation and suggesting ROS-mediated pathway reactivation.

Necroptosis is a dynamic process, with temporal changes in marker expression following IRI. Previous studies examining necroptosis marker expression reported pathway activation between 3 and 12 h post-reperfusion [12]; however, these studies were limited to male mice and assessed only at the mRNA level. In contrast, our study utilized protein-level analysis via Western blot to quantify RIPK1, RIPK3, and MLKL in both sexes and immunofluorescence staining to detect the spatial pattern of necroptosis markers across multiple time points. We also demonstrate that female mice exhibit delayed and attenuated necroptotic signaling, with MLKL activation not reaching significant levels until 12 h post-reperfusion—and at much lower intensity than in males. The spatial distribution of MLKL in male kidneys further evolved over time, transitioning from cytoplasmic to apical localization and intraluminal deposition, suggesting sustained epithelial injury not observed in females. By 72 h, MLKL levels had declined substantially in both sexes but were nearly undetectable in females.

Consistent with previous studies, male mice sustained greater kidney injury and exhibited higher serum creatinine and tubular damage scores compared to females. To our knowledge, this is the first demonstration that females exhibit delayed/attenuated necroptosis, with significant activation only after 12 h at reduced levels. This reduced response during the critical 3–12 h window contributes to the improved structural and functional outcomes observed in females.

Sexual dimorphism in AKI has been reported across various etiologies, with females—especially premenopausal—generally exhibiting less severe disease [33,34,35]. Multiple factors, including sex hormones, influence this disparity. Estrogen is often protective, while testosterone is linked with increased susceptibility to injury. Our data support this notion, as ovariectomy in female mice partially abolished the sex difference in necroptosis marker expression and injury severity. However, ovariectomy did not fully replicate the male phenotype, which could be due to preexisting differences in the male hormones. Additional influences, including genetic, immunologic, chromosomal complement, developmental factors, or non-gonadal hormone sources, may also shape sex-specific responses to AKI [36,37,38]. Sex-specific differences in immune system function have been documented, with females generally exhibiting stronger innate and adaptive immune responses. This enhanced immunity could contribute to differential outcomes post-injury by influencing clearance of damaged cells and tissue remodeling [38]. Furthermore, non-hormonal systemic factors such as differences in metabolism, vascular function, and oxidative stress response may contribute to the sex-specific outcomes observed [39,40]. Collectively, these multifaceted influences underscore the importance of considering a broader biological context beyond female sex hormones when investigating sex differences. Future research integrating genomic, immunologic, chromosome complement, and metabolic approaches will be critical to fully elucidate the mechanisms driving these disparities and to inform sex-specific therapeutic strategies.

Despite these insights, our study has limitations. It was restricted to a bilateral IRI model and may not capture necroptosis dynamics in other AKI etiologies such as sepsis or nephrotoxicity. Although we examined multiple timepoints, higher temporal resolution could further refine our understanding of necroptosis kinetics. Moreover, our data does not directly establish causality between necroptosis activation and injury severity, which warrants further investigation using pathway-specific inhibitors or knockout models. The partial abolition of sex differences observed in the ovariectomy group was based on a limited set of parameters. Future studies incorporating expanded analyses, including sex hormone supplementation, are needed to validate and extend these findings and to better understand the role of hormones in sex-specific regulation of necroptosis, as suggested by our preliminary data.

In conclusion, our findings provide compelling evidence of sex-specific differences in necroptosis activation during IRI-induced AKI. Male mice exhibited earlier, stronger, and more prolonged activation of the RIPK1–MLKL axis, correlating with more severe renal injury and delayed recovery. In contrast, females showed a delayed and weaker necroptotic response, which may underlie their relatively better outcomes. These results highlight necroptosis as a promising therapeutic target in AKI and suggest that sex-specific treatment strategies may enhance clinical outcomes. Moreover, necroptosis markers such as p-RIPK1 and p-MLKL could serve as valuable biomarkers for patient stratification and treatment response. Future work should explore the efficacy of necroptosis inhibitors in both sexes and assess how hormonal and non-hormonal factors modulate this pathway to advance personalized AKI therapies.

## Data Availability

All the data and materials supporting the findings of this study are available within the article. Further inquiries can be directed at the corresponding author.

## Supplementary Materials

The following supporting information can be downloaded at: https://www.mdpi.com/article/10.3390/biomedicines13092085/s1.

## References

[1] Pefanis A., Ierino F.L., Murphy J.M., Cowan P.J. (2019). Regulated necrosis in kidney ischemia-reperfusion injury. Kidney Int..

[2] Al-Jaghbeer M., Dealmeida D., Bilderback A., Ambrosino R., Kellum J.A. (2018). Clinical Decision Support for In-Hospital AKI. J. Am. Soc. Nephrol..

[3] Zarjou A., Agarwal A. (2011). Sepsis and acute kidney injury. J. Am. Soc. Nephrol..

[4] Bonventre J.V., Yang L. (2011). Cellular pathophysiology of ischemic acute kidney injury. J. Clin. Investig..

[5] Li C., Yu Y., Zhu S., Hu Y., Ling X., Xu L., Zhang H., Guo K. (2024). The emerging role of regulated cell death in ischemia and reperfusion-induced acute kidney injury: Current evidence and future perspectives. Cell Death Discov..

[6] Sun L., Wang H., Wang Z., He S., Chen S., Liao D., Wang L., Yan J., Liu W., Lei X. (2012). Mixed Lineage Kinase Domain-like Protein Mediates Necrosis Signaling Downstream of RIP3 Kinase. Cell.

[7] Liu S., Liu H., Johnston A., Hanna-Addams S., Reynoso E., Xiang Y., Wang Z. (2017). MLKL forms disulfide bond-dependent amyloid-like polymers to induce necroptosis. Proc. Natl. Acad. Sci. USA.

[8] He S., Wang X. (2018). RIP kinases as modulators of inflammation and immunity. Nat. Immunol..

[9] He S., Wang L., Miao L., Wang T., Du F., Zhao L., Wang X. (2009). Receptor Interacting Protein Kinase-3 Determines Cellular Necrotic Response to TNF-α. Cell.

[10] Cho Y.S., Challa S., Moquin D., Genga R., Ray T.D., Guildford M., Chan F.K.-M. (2009). Phosphorylation-Driven Assembly of the RIP1-RIP3 Complex Regulates Programmed Necrosis and Virus-Induced Inflammation. Cell.

[11] Kearney C.J., Martin S.J. (2017). An Inflammatory Perspective on Necroptosis. Mol. Cell.

[12] Pefanis A., Bongoni A.K., McRae J.L., Salvaris E.J., Fisicaro N., Murphy J.M., Ierino F.L., Cowan P.J. (2023). Dynamics of necroptosis in kidney ischemia-reperfusion injury. Front. Immunol..

[13] Linkermann A., Bräsen J.H., Himmerkus N., Liu S., Huber T.B., Kunzendorf U., Krautwald S. (2012). Rip1 (Receptor-interacting protein kinase 1) mediates necroptosis and contributes to renal ischemia/reperfusion injury. Kidney Int..

[14] Chen H., Fang Y., Wu J., Chen H., Zou Z., Zhang X., Shao J., Xu Y. (2018). RIPK3-MLKL-mediated necroinflammation contributes to AKI progression to CKD. Cell Death Dis..

[15] Neugarten J., Golestaneh L. (2022). Sex Differences in Acute Kidney Injury. Semin. Nephrol..

[16] Hosszu A., Fekete A., Szabo A.J. (2020). Sex differences in renal ischemia-reperfusion injury. Am. J. Physiol. Physiol..

[17] Wei J., Wang Y., Zhang J., Wang L., Fu L., Cha B.J., Buggs J., Liu R. (2019). A mouse model of renal ischemia-reperfusion injury solely induced by cold ischemia. Am. J. Physiol. Physiol..

[18] Wei J., Zhang J., Wang L., Jiang S., Fu L., Buggs J., Liu R. (2019). New mouse model of chronic kidney disease transitioned from ischemic acute kidney injury. Am. J. Physiol. Physiol..

[19] Luengo-Mateos M., González-Vila A., Caldas A.M.T., Alasaoufi A.M., González-Domínguez M., López M., González-García I., Barca-Mayo O. (2024). Protocol for ovariectomy and estradiol replacement in mice. STAR Protoc..

[20] Rowe A.A., Issioui Y., Johnny B., Wert K.J. (2023). Murine Orchiectomy and Ovariectomy to Reduce Sex Hormone Production. J. Vis. Exp..

[21] Wang L., Wang X., Jiang S., Wei J., Buggs J., Fu L., Zhang J., Liu R. (2018). Graft function assessment in mouse models of single- and dual-kidney transplantation. Am. J. Physiol. Physiol..

[22] Wang L., Song J., Wang S., Buggs J., Chen R., Zhang J., Wang L., Rong S., Li W., Wei J. (2017). Cross-sex transplantation alters gene expression and enhances inflammatory response in the transplanted kidneys. Am. J. Physiol. Ren. Physiol..

[23] Chen W., Wang L., Liang P., Mast J., Mathis C., Liu C.Y., Wei J., Zhang J., Fu L., Juncos L.A. (2022). Reducing ischemic kidney injury through application of a synchronization modulation electric field to maintain Na+/K+ -ATPase functions. Sci. Transl. Med..

[24] Mulay S.R., Linkermann A., Anders H.J. (2016). Necroinflammation in Kidney Disease. J. Am. Soc. Nephrol..

[25] Linkermann A., Stockwell B.R., Krautwald S., Anders H.-J. (2014). Regulated cell death and inflammation: An auto-amplification loop causes organ failure. Nat. Rev. Immunol..

[26] Neugarten J., Golestaneh L. (2019). Influence of Sex on the Progression of Chronic Kidney Disease. Mayo Clin. Proc..

[27] Müller V., Losonczy G., Heemann U., Vannay A., Fekete A., Reusz G., Tulassay T., Szabó A.J. (2002). Sexual dimorphism in renal ischemia-reperfusion injury in rats: Possible role of endothelin. Kidney Int..

[28] Cai Z., Jitkaew S., Zhao J., Chiang H.-C., Choksi S., Liu J., Ward Y., Wu L.-G., Liu Z.-G. (2013). Plasma membrane translocation of trimerized MLKL protein is required for TNF-induced necroptosis. Nat. Cell Biol..

[29] Zhao J., Jitkaew S., Cai Z., Choksi S., Li Q., Luo J., Liu Z.-G. (2012). Mixed lineage kinase domain-like is a key receptor interacting protein 3 downstream component of TNF-induced necrosis. Proc. Natl. Acad. Sci. USA.

[30] Hanna-Addams S., Liu S., Liu H., Chen S., Wang Z. (2020). CK1alpha, CK1delta, and CK1epsilon are necrosome components which phosphorylate serine 227 of human RIPK3 to activate necroptosis. Proc. Natl. Acad. Sci. USA.

[31] Wang H., Sun L., Su L., Rizo J., Liu L., Wang L.-F., Wang F.-S., Wang X. (2014). Mixed Lineage Kinase Domain-like Protein MLKL Causes Necrotic Membrane Disruption upon Phosphorylation by RIP3. Mol. Cell.

[32] Wang L., Du F., Wang X. (2008). TNF-α Induces Two Distinct Caspase-8 Activation Pathways. Cell.

[33] Satake A., Takaoka M., Nishikawa M., Yuba M., Shibata Y., Okumura K., Kitano K., Tsutsui H., Fujii K., Kobuchi S. (2008). Protective effect of 17β-estradiol on ischemic acute renal failure through the PI3K/Akt/eNOS pathway. Kidney Int..

[34] Kang K.P., Lee J.E., Lee A.S., Jung Y.J., Kim D., Lee S., Hwang H.P., Kim W., Park S.K. (2014). Effect of gender differences on the regulation of renal ischemia-reperfusion-induced inflammation in mice. Mol. Med. Rep..

[35] Hutchens M.P., Fujiyoshi T., Komers R., Herson P.S., Anderson S. (2012). Estrogen protects renal endothelial barrier function from ischemia-reperfusion in vitro and in vivo. Am. J. Physiol. Physiol..

[36] Sharma S., Eghbali M. (2014). Influence of sex differences on microRNA gene regulation in disease. Biol. Sex Differ..

[37] McDonough A.A., Foley T.S., Ralph D.L., Schwindt S., Soong J., Gaytan R.C., Lasaad S., Nelson J.W., Edwards A., Kleyman T.R. (2025). Effect of sex chromosome complement versus gonadal hormones on abundance of renal transporters. Am. J. Physiol. Physiol..

[38] Klein S.L., Flanagan K.L. (2016). Sex differences in immune responses. Nat. Rev. Immunol..

[39] Shen H., Holliday M., Sheikh-Hamad D., Li Q., Tong Q., Hamad C.D., Pan J.S. (2021). Sirtuin-3 mediates sex differences in kidney ischemia-reperfusion injury. Transl. Res..

[40] Rodríguez F., Nieto-Cerón S., Fenoy F.J., López B., Hernández I., Martinez R.R., Soriano M.J.G., Salom M.G. (2010). Sex differences in nitrosative stress during renal ischemia. Am. J. Physiol. Integr. Comp. Physiol..

